# Medea: An omics AI agent for therapeutic discovery

**DOI:** 10.64898/2026.01.16.696667

**Published:** 2026-01-20

**Authors:** Pengwei Sui, Michelle M. Li, Shanghua Gao, Wanxiang Shen, Valentina Giunchiglia, Andrew Shen, Yepeng Huang, Zhenglun Kong, Marinka Zitnik

**Affiliations:** 1 Department of Biomedical Informatics, Harvard Medical School, Boston, MA, USA; 2 The Ivan and Francesca Berkowitz Family Living Laboratory Collaboration at Harvard Medical School and Clalit Research Institute, Boston, MA, USA; 3 Department of Brain Sciences, Imperial College London, London, UK; 4 Centre for Neuroimaging Sciences, King’s College London, London, UK; 5 Kempner Institute for the Study of Natural and Artificial Intelligence, Harvard University, Allston, MA, USA; 6 Broad Institute of MIT and Harvard, Cambridge, MA, USA; 7 Harvard Data Science Initiative, Cambridge, MA, USA

## Abstract

AI agents promise to empower biomedical discovery, but realizing this promise requires the ability to complete transparent, long-horizon analyses using tools. Agents must make intermediate decisions explicit, and validate each decision and output against data and tool constraints as the analysis unfolds. We present Medea, an AI agent that takes an omics objective and executes a transparent multi-step analysis using tools. Medea comprises four modules: research planning with context and integrity verification, code execution with pre- and post-run checks, literature reasoning with evidence-strength assessment, and a consensus stage that reconciles evidence across datasets, tools, and literature. Medea uses 20 tools spanning single-cell and bulk transcriptomic datasets, cancer vulnerability maps, pathway knowledge bases, and machine learning models. We evaluate Medea across 5,679 analyses in three open-ended domains: target identification across five diseases and cell type contexts (2,400 analyses), synthetic lethality reasoning in seven cell lines (2,385 analyses), and immunotherapy response prediction in bladder cancer (894 patient analyses). In evaluations that vary large language models, tool sets, omics objectives, and agentic modules, Medea improves the performance of existing approaches by up to 46% for target identification, 22% for synthetic lethality, and 24% for immunotherapy response prediction, while maintaining low failure rates and calibrated abstention. Medea shows that verification-aware AI agents improve performance by producing transparent analyses, not simply more efficient workflows.

## Introduction

Omics datasets support target discovery [1], studies of disease biology [2], and biomarker discovery [3] by linking gene activity to cellular phenotypes [4–6]. Chemogenomics adds another data layer that connects cellular states to compound responses [7, 8], and genetic studies provide evidence for how perturbing a gene affects disease risk and presentation [9, 10]. When combined, these omics datasets can link molecular mechanisms to disease states and perturbations [11–14]. For example, a genetic association can prioritize a gene, single-cell profiles can localize its activity to a disease-relevant cell population, and chemogenomics can suggest compound classes that shift that disease state toward a reference state. These analyses can help decide which candidate target genes to test, in which cellular models or patient populations, and with which intervention classes.

AI agents are beginning to inform these decisions by translating therapeutic hypotheses into research plans, retrieving relevant data, and executing workflows [15–23]. However, many agents still rely on large language model (LLM)’s parametric memory instead of grounding intermediate results in omics datasets, or they follow fixed templates that limit adaptation across omics analyses [24]. Three gaps limit how these agents can support therapeutic discovery from omics data. First, agents often lose track of biological context over long-horizon analyses. They do not consistently carry forward the specified cell type, disease, and patient cohort, so they may apply single-cell tools to bulk questions or query pathway knowledge bases curated for unrelated tissues. These context slips can bias agents toward marker genes from abundant cell types rather than targets in the disease-relevant compartment [19, 21]. Second, agents rarely verify feasibility before and after execution. Pre-run checks do not test tool-dataset assumptions and statistical requirements, and post-run checks often stop at catching runtime errors. As a result, an analysis can run successfully but still be wrong, for example, when differential expression uses mismatched covariates that are propagated into downstream pathway ranking [22, 23]. Third, agents can struggle to reconcile evidence across datasets, often aggregating studies without screening for relevance [19, 20]. These gaps motivate the development of agents that preserve biological context, validate analyses as they run, and resolve potentially conflicting evidence across omics datasets and tools.

Addressing these gaps requires agents that ground conclusions in omics data by coordinating tools that retrieve, analyze, and interpret omics readouts. Many of the tools that agents call are machine learning models. Contextual embedding models, for example, represent genes in ways that depend on biological context, including the tissue, the cell type, and the cell state in which they are measured [25–28]. When paired with large biological datasets [29, 30] and literature [31], these models provide context-specific evidence that an agent can retrieve and integrate into its analysis. Perturbation models add a complementary class of tools by estimating how genetic or chemical interventions change gene expression and cellular phenotypes, allowing an agent to compare disease and perturbation signatures and prioritize interventions expected to impact disease states [32–35].

Here, we present Medea, an AI agent that takes an omics objective and executes a multi-step analysis with verification at each step. Medea comprises four modules: planning that specifies the biological context and checks plan integrity, execution that runs code with pre-run validation and post-run checks, literature reasoning that retrieves and screens studies for contextual relevance given the omics objective, and consensus module that reconciles evidence from tool outputs, literature, and the underlying language model, or abstains when evidence is insufficient. Medea operates over a tool space spanning single-cell and bulk transcriptomics, protein networks, pathway and ontology resources, and foundation models, and we evaluate it in three open-ended domains. In cell type specific target nomination, Medea prioritizes candidate targets in disease-relevant cell types rather than tissue-level averages. Across 2,400 analyses covering five diseases and primary cell types, Medea improves the accuracy of LLMs by up to 45.9% in rheumatoid arthritis and 32.9% in Sjögren’s Syndrome. In synthetic lethality reasoning, Medea integrates genetic interaction signals with pathway evidence to identify gene pairs whose combined inhibition is predicted to impair cancer cell viability. Across 2,385 analyses in seven cell lines, Medea improves accuracy by up to 21.7% in MCF7 and 13.9% in A549, with lower failure rates than the LLM alone. In immunotherapy response prediction, Medea links tumor-intrinsic and microenvironment programs to treatment responses by integrating signals related to antigen presentation, interferon signaling, and T-cell exhaustion. Across 894 analyses involving 298 patients, Medea achieves up to 23.9% higher accuracy than existing models. Ablations show complementary contributions of Medea’s modules: a literature-only configuration abstains in 79.1% of disease contexts, whereas an LLM-only configuration abstains in 1.8% of analyses but accounts for the largest share of errors; the full Medea achieves the best performance with the lowest failure rate.

## Results

### Medea agent for verified omics reasoning

**Figure 1:**
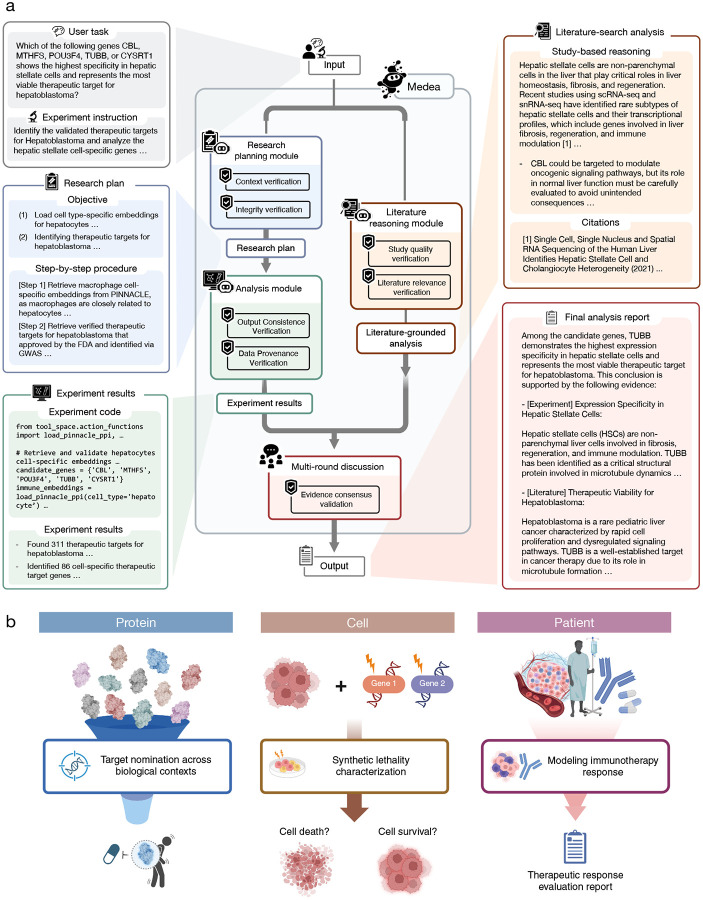
Overview of Medea and open-ended evaluation domains. **(a)** Medea takes an omics objective and an optional experiment instruction, produces a research plan, executes omics analyses using tools, retrieves and screens literature, and reconciles evidence to return a final report or calibrated abstention. Medea consists of four modules: ResearchPlanning (context and integrity verification for plan construction), Analysis (tool execution with pre-run checks and post-run verification), LiteratureReasoning (literature retrieval with relevance and evidence-strength assessment), and MultiRoundDiscussion (evidence reconciliation across module outputs). **(b)** Medea is evaluated on three domains: cell type specific target nomination, synthetic lethality reasoning in cell lines, and immunotherapy response prediction from patient tumor transcriptomes and clinical data.

**Figure 2:**
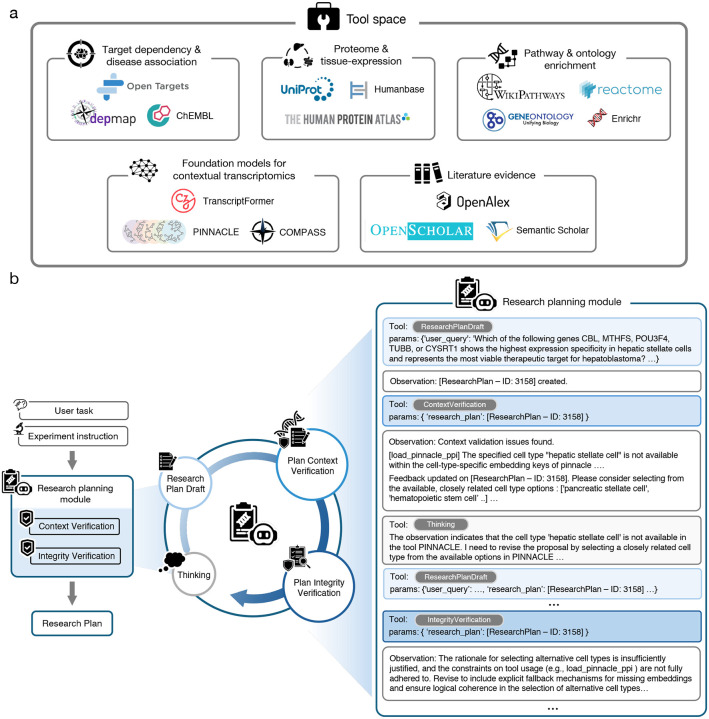
Tool space and verified planning in Medea. **(a)** Medea uses a global tool space (Methods 1.1) with resources about therapeutic targets, disease associations, proteomics, and tissue expression; tools to perform gene set enrichment and pathway analyses; machine learning models for single-cell and bulk omics; and tools for literature retrieval. **(b)** Given an omics objective (user instruction) and an optional experiment instruction, the ResearchPlanning module (Methods 1.2) generates a multi-step analysis plan. Context verification checks tool and data compatibility with the research objective. Integrity verification audits the research plan’s feasibility, completeness, and logical consistency.

Medea takes as input an omics-based objective specified as a natural language instruction and an optional experiment plan. To support different levels of user expertise, Medea accepts both high-level plans and detailed research plans. Medea couples agentic modules with tool use (Figure 1a) and can invoke any of 20 tools (Methods 1.1, Figure 2a) to execute multi-step analyses, returning a report grounded in outputs from machine learning models [26, 27, 36], multimodal datasets [37–46], and literature [47–49].

Medea orchestrates four modules: ResearchPlanning (Methods 1.2), Analysis (Methods 1.3), LiteratureReasoning (Methods 1.4), and MultiRoundDiscussion (Methods 1.5). The ResearchPlanning module transforms an omics-based objective into an executable computational experiment plan, decomposing complex instructions into tractable subtasks, selecting appropriate tools (e.g., databases, machine learning models) for each step, and verifying plan integrity by assessing specificity, technical feasibility, consistency, and logical validity [50–53]. The Analysis module translates the plan into tool calling analyses (databases/APIs, machine learning models, and other agents) and enforces verification through pre-execution validation (syntax and dependency checks), sandboxed execution with automated error trapping, and post-execution verification (data provenance auditing [54]) to ensure outputs remain aligned with the plan and the omics objective. The LiteratureReasoning module retrieves literature to produce an evidencegrounded response, using Semantic Scholar [47] and OpenAlex [48] for retrieval, performing relevance screening, and using OpenScholar [49] to generate literature summaries. Medea initiates reasoning by querying the backbone LLM to derive a preliminary response from parametric knowledge and then instantiating a multi-round deliberative process [55] in which a panel of LLMs reviews and reconciles outputs from the Analysis, LiteratureReasoning, and backbone LLM to form a consensus response with calibrated abstention when evidence is insufficient. In evaluations, Medea can activate any subset of modules and tools to complete omics objectives, a capability we evaluate across three open-ended domains: cell type specific target nomination, synthetic lethality reasoning, and immunotherapy response prediction.

### Benchmark for cell type specific target nomination

**Figure 3:**
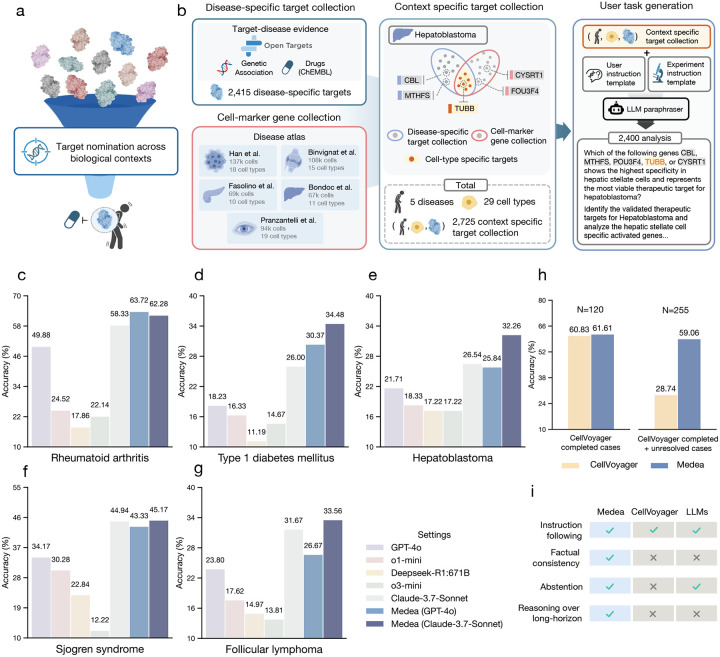
Cell type specific target nomination benchmark. **(a)** Given a disease, a cell type, and a set of candidate genes, Medea executes the omics objective of nominating the most supported target for the specified context by integrating evidence from databases, single-cell foundation models, and the scientific literature (Methods 2). **(b)** Constructing the benchmark dataset of context-specific targets across five diseases and multiple primary cell types leverages single-cell atlases and resources about therapeutic targets and disease-gene associations. **(c-h)** Performance of Medea compared to five LLMs and a single-cell computational biology agent, CellVoyager [21], across disease and cell type contexts. **(i)** Qualitative comparison of capabilities relevant to this benchmark, including instruction following, factual consistency checks, calibrated abstention, and long-horizon multi-step reasoning.

A target’s therapeutic potential reflects how likely a gene or protein is a useful point of intervention, meaning that perturbing it produces a meaningful disease-relevant effect with an acceptable safety profile. This potential depends on biological context, including the tissue, cell type, and cellular state in which the target is active [56]. When this context is ignored, on-target efficacy can be reduced and the risk of off-target toxicity can increase [57–59]. Yet translating context specificity into computational target nomination remains challenging [26, 60, 61]. Many machine learning approaches are trained on bulk tissue or established cell lines, and therefore lack the resolution needed to assess therapeutic hypotheses at the level of specific cell types [62, 63]. To evaluate whether agents can perform this type of therapeutic analysis, we construct a benchmark for cell type specific target nomination (Figure 3a).

The benchmark spans 29 cell types across five diseases: rheumatoid arthritis (RA) [64], type 1 diabetes mellitus (T1DM) [65], Sjögren’s syndrome (SS) [66], hepatoblastoma (HB) [67], and follicular lymphoma (FL) [68] (Figure 3b; Methods 2). For each disease, we define cell type specific targets by retrieving single-cell transcriptomic atlases from healthy individuals and patients, and identifying differentially expressed genes per cell type between healthy and disease states. We then filter candidates for prior evidence of involvement in disease mechanisms [69] or association with approved or clinical candidate drugs (via ChEMBL) [70]. We define negative samples as genes that are not differentially expressed in patients compared to healthy individuals or that lack genetic and clinical trial support. For each disease–cell-type context, we generate 60 analyses in which the agent receives a disease, a cell type, and five candidate genes, yielding 2,400 analyses in total (420 across 7 cell types for RA, 600 across 9 cell types for T1DM, 360 across 6 cell types for SS, 180 across 3 cell types for HB, and 840 across 14 cell types for FL). In each analysis, the agent or model selects the gene with the strongest evidence of therapeutic potential in the specified disease and cell type and provides a rationale.

The benchmark is challenging in three ways. First, it requires completing an omics-based objective at cell type resolution within a specified disease context, rather than reasoning at the bulk tissue level. Second, it links single-cell differential expression with orthogonal evidence from human genetics and clinical trials, so methods must integrate multiple evidence sources rather than relying on gene expression alone. Third, the 2,400 analyses span immune and malignant diseases and diverse cell types, testing whether methods generalize across contexts rather than optimizing for a single indication. We evaluate models and agents using per-analysis accuracy, defined as the fraction of analyses in which the correct gene is nominated among the five candidates. For agents that can abstain when confidence is low, we additionally report the abstention rate and accuracy conditioned on non-abstention to quantify both coverage and correctness of performance.

### Cell type specific target nomination

Medea is evaluated against five LLMs and a computational biology agent, CellVoyager [21], on the omics objectives of nominating therapeutic targets in disease-relevant cell types for rheumatoid arthritis, type 1 diabetes mellitus, Sjögren’s Syndrome, hepatoblastoma, and follicular lymphoma. For each of the 2,400 analyses (omics objectives), Medea provides an answer and a report that summarizes the rationale for each candidate target in a cell type and disease context.

Medea performs competitively against LLMs, reasoning models, and CellVoyager in nominating cell type specific therapeutic targets (Figure 3). Medea outperforms LLMs by up to 45.9% in accuracy for rheumatoid arthritis (*p*-value < 0.0001 using McNemar’s test [71]; Figure 3c); 23.3% for type 1 diabetes mellitus (*p*-value < 0.0001; Figure 3d); 33.0% for Sjögren’s Syndrome (*p*-value < 0.0001; Figure 3e); 15.0% for hepatoblastoma (*p*-value < 0.0001; Figure 3f); and 19.8% for follicular lymphoma (*p*-value < 0.0001; Figure 3g). We also evaluate Medea with GPT-4o or Claude 3.7 Sonnet as the LLM backbone.

Medea consistently outperforms its backbone LLM, which is defined as the underlying language model used on its own without the ResearchPlanning, Analysis, or LiteratureReasoning modules. For example, Medea (GPT-4o) and Medea (Claude 3.7 Sonnet) are 12.1% (*p*-value < 0.0001) and 8.5% (*p*-value = 0.0004) more accurate than GPT-4o and Claude 3.7 Sonnet alone, respectively, on the omics objectives for type 1 diabetes mellitus target nomination (Figure 3d). These results show that integrating verification-aware modules and tools improves target nomination across disease and cell type contexts.

Since CellVoyager is designed to reproduce single-cell analyses from scientific papers [21], we evaluate whether it can nominate therapeutic targets for rheumatoid arthritis [26]. We compare CellVoyager against Medea (Claude 3.7 Sonnet) using the global tool space (Section 1.1). Because CellVoyager produces lengthy multimodal outputs and exhibits high failure rates, a human expert reviews and scores all CellVoyager outputs. Across 420 analyses, CellVoyager completes 28.6% (120 out of 420). On these 120 completed analyses, CellVoyager is 0.8% less accurate than Medea (Figure 3h). In 39.3% of analyses (165 out of 420), CellVoyager fails to nominate any target due to errors during data preprocessing; on the remaining 255 analyses, Medea is 30.3% (or 2.1 times) more accurate than CellVoyager (Figure 3h). We also find that CellVoyager does not enforce factual consistency [72–74] (Figure 3i; Supplementary Note 6); for example, it often hallucinates gene names. Finally, while LLMs can be instructed to abstain when uncertain, CellVoyager is not designed to abstain [75–77] (Figure 3i), and struggles with long-horizon reasoning [50, 78, 79] because it only interprets the output of the most recent Jupyter cell (Figure 3i). In contrast, Medea maintains factual consistency at each step of the analysis, can abstain when evidence is insufficient, and completes analyses that require multi-step reasoning over long horizons, which correspond to higher accuracy and lower failure rates on the benchmark.

### Context verification improves cell type level performance

We next assess Medea’s ability to reason about candidate targets within the user-specified cell type context, which is critical for therapeutic efficacy and safety. Completing this omics-based objective requires correctly identifying the stated context and retrieving context-appropriate evidence [19]. Closely related cell types can have distinct roles; for example, naïve CD4^+^ *αβ* T cells versus effector memory CD4^+^ *αβ* T cells and naïve CD8^+^ *αβ* T cells [80, 81]. However, LLMs often miss such distinctions [19, 24] and may default to higher-level lineages, such as CD4^+^ or CD8^+^ T cells. Medea is designed to perform context verification [82, 83] so that intermediate decisions remain consistent with the specified cell type and disease context. To quantify the contribution of context verification, we stratify Medea’s performance across the cell type and disease contexts in the target nomination benchmark (Methods 2).

**Figure 4:**
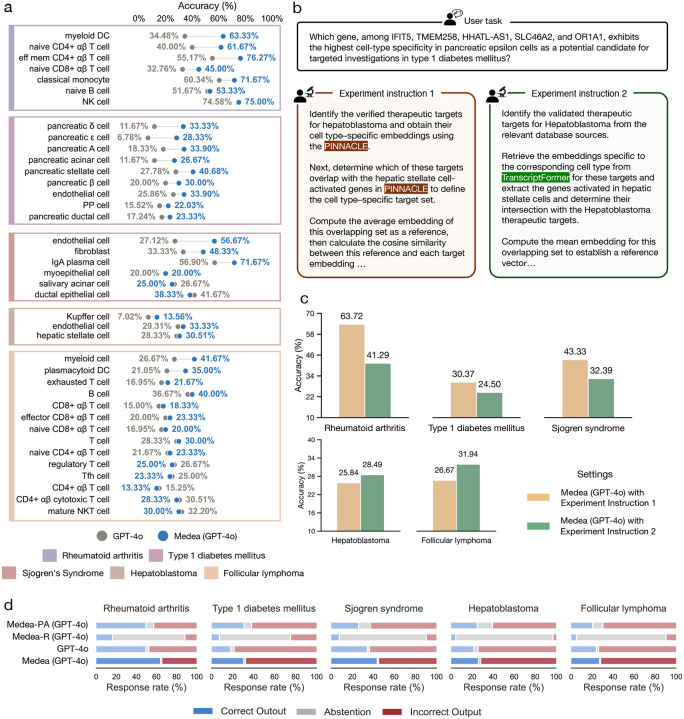
Medea performs cell type specific target nomination through multimodal agentic reasoning. **(a)** Accuracy of Medea (GPT-4o) and its backbone LLM (GPT-4o), stratified by disease and cell type, on the cell type specific target nomination benchmark. **(b-c)** Tool-constrained experiments in which Medea is instructed to use a specific single-cell foundation model (PINNACLE [26] or TranscriptFormer [27]) for linking the candidate genes to disease-relevant cell types, evaluating the impact of tool choice on performance. **(d)** Agent module ablations that quantify how planning, tool execution, and literature reasoning by Medea contribute to correctness and calibrated abstention. Medea-PA denotes the configuration that activates the ResearchPlanning and Analysis modules (Methods 1.2–1.3) to execute tool-based reasoning without literature synthesis. Medea-R denotes the configuration that activates only the LiteratureReasoning module (Methods 1.4). Medea denotes the full agent configuration that activates all four modules (i.e., ResearchPlanning, Analysis, LiteratureReasoning, MultiRoundDiscussion).

Medea performs comparably or better than GPT-4o in diverse cell type and disease contexts (Figure 4a). Medea boosts the accuracy of predicting therapeutic targets for rheumatoid arthritis in myeloid dendritic cells by 28.9%, naïve CD4^+^ *αβ* T cells by 21.7%, effector memory CD4^+^ *αβ* T cells by 21.1%, and naïve CD8^+^ *αβ* T cells by 12.2%. Performance gains in nominating therapeutic targets for rheumatoid arthritis within these cell types are meaningful. The localization of certain myeloid dendritic cells in the synovium of patients with rheumatoid arthritis has been associated with immune homeostasis [84]. As different subsets of CD4+ [85] and CD8^+^ T cells [86] contribute uniquely to the pathogenesis of rheumatoid arthritis, it is important to consider granular subtypes (e.g., naïve versus effector memory CD4^+^ *αβ* T cells) for nominating therapeutic targets. Medea and GPT-4o perform comparably on naïve B cells and natural killer (NK) cells. For type 1 diabetes mellitus, Medea consistently outperforms GPT-4o on all nine cell type contexts, with performance gains of up to 21.7%. For Sjögren’s Syndrome, Medea yields improved average accuracies by 29.6% in endothelial cells, 15% in fibroblasts, and 14.8% in IgA plasma cells. Endothelial cells and fibroblasts contribute to the recruitment of or interact with lymphocytes in salivary glands, which are particularly affected by Sjögren’s Syndrome, respectively [87]. Since IgA plasma cells are enriched in patients with Sjögren’s Syndrome, they serve as one of the histopathological features for diagnosis [88, 89] and a potential target for treating systemic autoimmune rheumatic diseases [90]. Medea’s accuracy doubles (7.0% vs. 13.6%) when nominating therapeutic targets for hepatoblastoma in Kupffer cells, which are the liver’s first line of defense [91]. For follicular lymphoma, Medea achieves 15.0% and 13.9% higher average accuracy in myeloid cells and plasmacytoid dendritic cells than GPT-4o, respectively. Myeloid cells are manipulated by tumor cells to promote tumor angiogenesis, cell invasion, and metastasis [92], and the activation of plasmacytoid dendritic cells can boost innate and adaptive cancer immunity [93]. These findings indicate that Medea identifies the disease-relevant cell type context, retrieves and executes context-appropriate tools, and reasons consistently within the specified context.

### Dissecting contributions of Medea’s agentic modules

To quantify how Medea achieves cell type specific target nomination, we perform ablation analyses of its agentic modules. Because Medea can invoke different tools and agents, we isolate the contribution of each component by restricting Medea to specific subsets of tools and/or modules and measuring its accuracy on the omics objectives of nominating cell type specific therapeutic targets across five disease contexts (Figure 4b–d).

To assess how tool choice affects performance, we instruct Medea to use either PINNACLE [26] or TranscriptFormer [27] for the omics objectives of nominating cell type specific therapeutic targets (Figure 4b). Neither tool consistently outperforms the other when used by Medea. With PINNACLE, Medea performs best for rheumatoid arthritis, type 1 diabetes mellitus, and Sjögren’s Syndrome. With TranscriptFormer, Medea performs better or comparably to the best LLM for hepatoblastoma (Figure 3f) and follicular lymphoma (Figure 3g), respectively. These results show that different disease contexts benefit from different tools, motivating agent access to a suite of complementary tools for completing omics objectives.

Based on the omics-based objective, Medea can activate different combinations of modules to complete the analysis. To quantify module contributions, we compare: pretrained knowledge from the backbone LLM only (GPT-4o), the ResearchPlanning and Analysis modules (Medea-PA), the LiteratureReasoning module only (Medea-R), and the full agent (Medea). Using all modules generally yields the highest accuracy and the lowest abstention rates (Figure 4d), consistent with reconciling complementary evidence through the MultiRoundDiscussion module when individual pathways are incomplete or conflicting. For example, because the literature on cell type specific therapeutic targets is limited, Medea-R abstains most frequently, with an average abstention rate of 77.6% across five diseases, whereas the backbone LLM abstains less (1.8% on average). However, the backbone LLM also produces the highest rate of incorrect nominations across disease contexts: 49.1% in rheumatoid arthritis, 80.0% in type 1 diabetes mellitus, 65.3% in Sjögren’s Syndrome, 76.1% in hepatoblastoma, and 75.5% in follicular lymphoma. These ablations show that Medea’s modules act synergistically to improve its performance on completing omics objectives.

### Inferring synthetic lethality in cellular contexts

**Figure 5:**
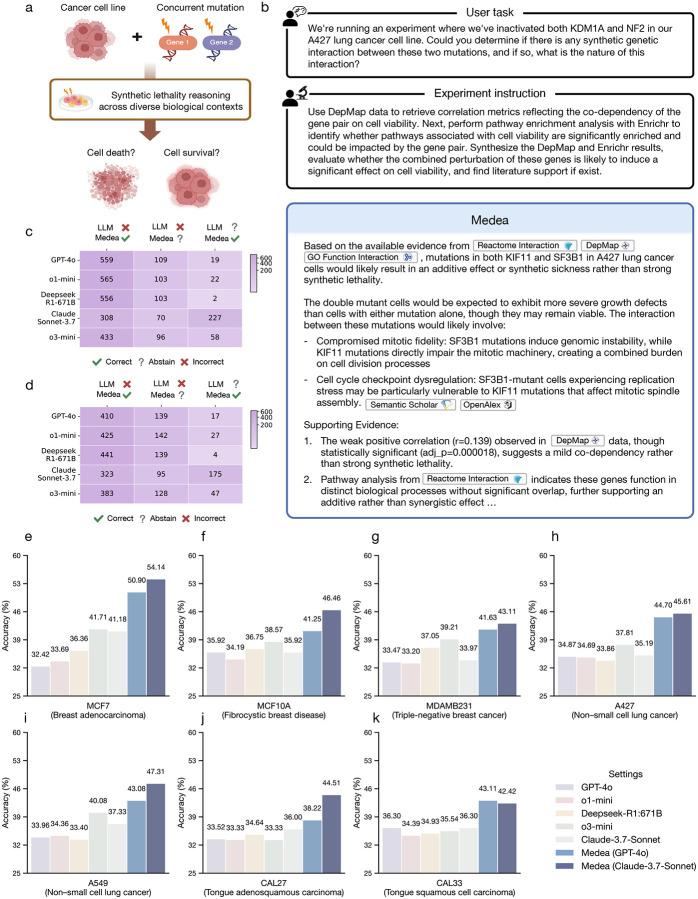
Agent reasoning about genetic interactions that affect cellular viability. **(a)** Given a pair of genes and a cell line context, Medea completes the omics objective of assessing whether the genes’ combined perturbation is consistent with reduced viability beyond single-gene effects (synthetic lethality; Methods 3). **(b)** Medea takes a natural language description of the omics objective (user instruction) and an optional experiment instruction, and produces an evidence-grounded response using tools (e.g., genetic dependency maps, pathway enrichment analyses) and literature evidence. **(c-d)** Number of analyses where the backbone LLM is incorrect but Medea is correct or abstains, and where the backbone LLM abstains but Medea is correct. **(e-k)** Performance of Medea and five LLMs on synthetic lethality reasoning across seven cell lines.

We next evaluate Medea on the omics objectives of inferring synthetic lethality in a given cellular context (Figure 5a) [94, 95]. Synthetic lethality occurs when perturbing two genes together reduces cellular viability substantially more than perturbing either gene alone, reflecting a synergistic interaction between the perturbations. Synthetic lethality can be exploited to design therapies that selectively kill cancer cells while limiting toxicity to normal cells [94, 95]. Using a multilineage CRISPR screen that identified synthetic lethal interactions in seven cell lines [96], we construct an open-ended benchmark for synthetic lethality reasoning (Methods 3.1). The benchmark includes 265 synthetic lethal gene pairs and 1,591 non-synthetic lethal pairs across seven cell lines: MCF7 (breast adenocarcinoma), MCF10A (fibrocystic breast disease), MDAMB231 (triple-negative breast cancer), CAL27 (tongue adenosquamous carcinoma), CAL33 (tongue squamous cell carcinoma), A549 (non-small cell lung cancer), and A427 (non-small cell lung cancer).

Medea can activate all modules, agents, and tools, including tools that analyze DepMap gene co-dependency scores from CRISPR-Cas9 essentiality screens in cancer cells [97], biological pathways [37, 40, 98, 99], and molecular function datasets [41, 100, 101] (Methods 1.1). Across all seven cell line contexts, Medea achieves higher accuracy than LLMs and reasoning models on the omics objectives of synthetic lethality inference (Figure 5c–i). Compared to LLMs, Medea achieves stronger accuracy by up to 21.7% in MCF7 (*p*-value = 0.0002 using McNemar’s test [71]; Figure 5c), 12.3% in MCF10A (*p*-value = 0.0012; Figure 5d), 9.9% in MDAMB231 (*p*-value 0.0220; Figure 5e), 11.2% in CAL27 (*p*-value = 0.0145; Figure 5f), 8.7% in CAL33 (*p*-value = 0.0018; Figure 5g), 13.9% in A549 (*p*-value < 0.0001; Figure 5h), and 11.8% in A427 (*p*-value < 0.0001; Figure 5i). Further, we observe performance gains compared to LLMs regardless of Medea’s LLM backbone (GPT-4o or Claude 3.7 Sonnet). While Medea (GPT-4o) and Medea (Claude 3.7 Sonnet) have comparable performance in the MDAMB231 (Figure 5e), CAL33 (Figure 5g), and A427 (Figure 5i) cell line contexts, Medea (Claude 3.7 Sonnet) yields higher accuracy in MCF7 by 3.2% (Figure 5c), MCF10A by 5.2% (Figure 5d), CAL27 by 6.3% (Figure 5f), and A549 by 4.2% (Figure 5h).

### Medea corrects errors and abstentions of LLMs

We next test whether verification enables Medea to revise intermediate steps and convert unreliable LLM outputs into more reliable conclusions for omics objectives, which is important when prioritizing candidate perturbations for follow-up. We therefore focus on analyses where Medea is correct while an LLM used alone is incorrect or abstains (Figure 5j–k). Medea (GPT-4o) and Medea (Claude 3.7 Sonnet) correctly infer the viability outcome in at least 323 (13.5%) and 308 (12.9%) analyses, respectively, for which GPT-4o, o1-mini, Deepseek R1 671B, Claude 3.7 Sonnet, and o3-mini are incorrect (Figure 5j). In addition, for up to 175 (7.3%) and 227 (9.5%) gene pairs where an LLM abstains, Medea (GPT-4o) and Medea (Claude 3.7 Sonnet) correctly identify the interaction (Figure 5j). In these cases, Medea uses literature and data tools to re-evaluate the gene pair, check consistency with reported genetic screens, and prioritize interactions supported by convergent evidence, refining synthetic lethality inference beyond parametric knowledge alone.

Because false positives and false negatives can trigger unnecessary wet-lab experiments or missed therapeutic opportunities [95], we next analyze cases where an LLM is incorrect but Medea abstains. Medea (GPT-4o) and Medea (Claude 3.7 Sonnet) abstain in up to 141 (5.9%) and 109 (4.6%) analyses, respectively, when an LLM makes an incorrect prediction (Figure 5j). Although Claude 3.7 Sonnet has a relatively high abstention rate when used alone, it does not degrade the performance of Medea (Claude 3.7 Sonnet), indicating that Medea’s abstention behavior is not solely determined by the LLM backbone. Instead, abstention reflects the joint effect of the ResearchPlanning, Analysis, and LiteratureReasoning modules, which surface internal inconsistencies and choose not to commit to a synthetic lethal prediction (Figure 4d). For example, Medea abstains when quantitative signals are weak or when pathway and empirical evidence do not corroborate the interaction (Supplementary Note 7). These results show that Medea can correct LLM errors and selectively abstain in uncertain cases of synthetic lethality inference.

### Personalized treatment response prediction from tumor transcriptomes

**Figure 6:**
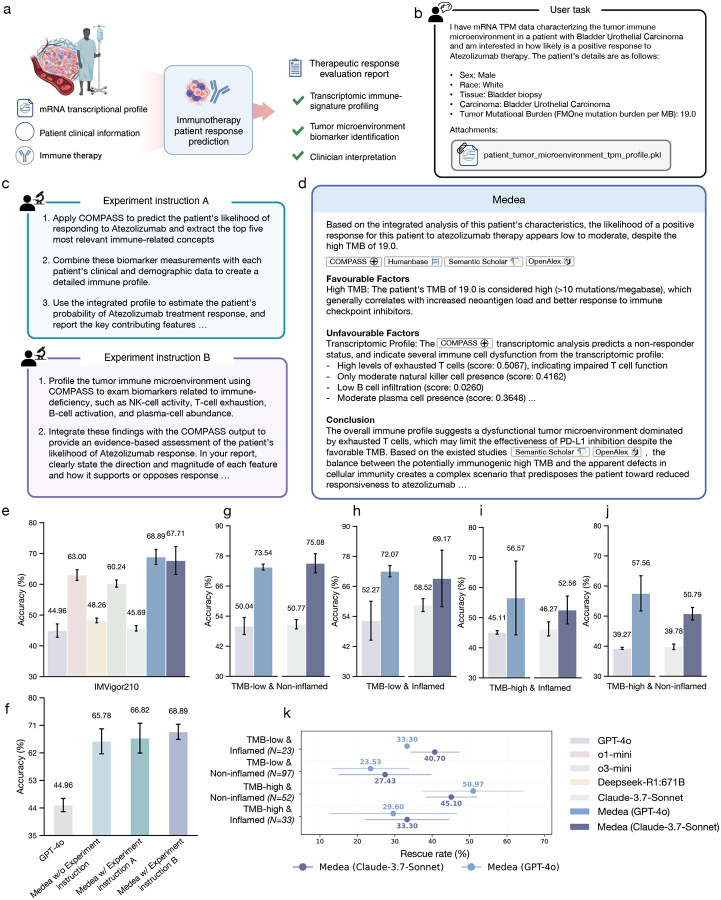
Multimodal reasoning for immunotherapy response prediction. **(a)** Given a patient’s clinical profile and tumor transcriptomic readout, Medea completes the omics objective of predicting immunotherapy response and returns a report with supporting evidence (Methods 4). **(b)** Multimodal inputs to Medea comprise clinical covariates and a tumor transcriptomic profile. **(c)** Medea accepts broad or detailed experiment instructions. **(d)** Medea grounds its report in evidence from an omics machine learning model (COMPASS [36]), tissue-specific protein networks [37], and literature [47, 48]. **(e)** Performance of Medea and five LLMs on predicting immunotherapy response. **(f)** Sensitivity of Medea (GPT-4o) and its backbone LLM (GPT-4o) to prompt variants: omics objective only, omics objective with a broad experiment instruction, and omics objective with a detailed experiment instruction. **(g-j)** Stratified performance of Medea (GPT-4o) and Medea (Claude 3.7 Sonnet) and their corresponding backbone LLMs (GPT-4o or Claude 3.7 Sonnet) on four clinical subgroups in the IMvigor210 cohort [105]. **(k)** Rescue analysis on IMvigor210 [105] patients for whom Medea correctly predicts the response label despite the incorrect prediction by the omics machine learning model [36]. Rescue rates are stratified by clinical subgroup.

We apply Medea to multimodal patient data, including clinical and transcriptomic profiles, to predict personalized response to immunotherapy (Figure 6a–b). Immunotherapy aims to treat cancer by activating the patient’s immune system against tumor cells; immune checkpoint inhibitors block proteins, such as CTLA-4 and PD-1, on T cells so that these cells can recognize and kill tumor cells [102]. However, biomarkers of treatment response are limited [103], and established ones, including tumor mutational burden (TMB) and features of the tumor microenvironment, are not consistently reliable predictors [104]. Using the IMvigor210 patient cohort [105], we construct an open-ended benchmark of 298 patients with bladder urothelial carcinoma treated with atezolizumab monotherapy (Methods 4). For each patient, Medea generates a report that includes a predicted responsiveness score and a rationale grounded in evidence relevant to the patient’s tumor transcriptomic profile, tumor microenvironment, and clinical profile (Figure 6a).

Medea can activate all modules and tools, including those that query COMPASS, an interpretable machine learning model for immunotherapy response prediction [36]. Medea reasons over the biologically grounded signatures learned by COMPASS to explain its prediction, including immune biomarkers, and combines these signals with evidence from other tools to generate the final report (Figure 6a). We evaluate Medea under three experiment instruction settings: without explicit experiment instructions, with broad guidance (experiment instruction A), and with detailed step-by-step constraints (experiment instruction B) (Figure 6c). While experiment instructions A and B both direct Medea to use COMPASS for analyzing the patient’s transcriptomic profile, experiment instruction A asks Medea to interpret the top five immune-related concepts ranked by COMPASS whereas experiment instruction B specifies the concepts to interpret.

Under experiment instruction B, Medea outperforms LLMs in predicting treatment response (Figure 6d). Medea achieves up to 23.9% higher accuracy than LLMs (*p*-value < 0.0001 using McNemar’s test [71]). Consistent with cell type specific target nomination and synthetic lethality (Figures 3–5), changing the underlying LLM used by Medea does not substantially affect performance on treatment response prediction (Figure 6e). In addition, varying or omitting the experiment instruction yields comparable performance, and Medea continues to outperform LLMs across these settings (Figure 6f). These results show that Medea is robust to differences in instruction specificity while completing the omics objectives of predicting treatment response from patients’ tumor transcriptomes.

### Interpreting treatment response from TMB and microenvironment

Immunotherapy response varies substantially across patients and tumor states [104, 106]. Tumor mutational burden (TMB) is often associated with improved response [104], but response also depends on features of the tumor microenvironment [107]. For instance, high TMB together with an inflamed microenvironment is more consistent with response than high TMB with a non-inflamed microenvironment [104]. Given this dependence on multiple interacting factors, we evaluate whether Medea can extract and reason over evidence about both TMB and the tumor microenvironment when inferring immunotherapy response.

We evaluate Medea (GPT-4o) and Medea (Claude 3.7 Sonnet) against their LLM backbones on treatment response prediction for patients across four subgroups: low TMB and non-inflamed (*N* = 97, with 16 responder and 81 non-responders; Figure 6g), low TMB and inflamed (*N* = 23, with 2 responders and 21 non-responders; Figure 6h), high TMB and inflamed (*N* = 33, with 15 responders and 18 non-responders; Figure 6i), and high TMB and non-inflamed (*N* = 64, with 25 responders and 39 non-responders; Figure 6j) tumor microenvironment. Medea achieves the highest accuracy across all groups. In contrast, standalone GPT-4o and Claude 3.7 Sonnet perform near random chance; for example, they tend to predict by default that patients with high TMB are responders. Further, Medea complements tool outputs by recovering false predictions. Across the four clinical subgroups, Medea correctly predicts response for patients that COMPASS misclassifies (Figure 6k), with rescue rates of up to 50.9% in challenging settings such as patients with high TMB and non-inflamed microenvironments. These results indicate that integrating transcriptomic, clinical, and mutational profiles with literature evidence improves treatment response inference, and that Medea benefits from agentic modules that combine tumor microenvironment from transcriptomes (via analysis of a patient’s transcriptomic profile by the COMPASS tool [36]) with TMB and other biomarker features (via literature retrieval tools) (Supplementary Notes 1–5).

To illustrate how Medea supports treatment response prediction, we present two patient vignettes from the IMvigor210 cohort [105] treated with atezolizumab. For each patient, Medea integrates clinical variables, tumor mutational burden, and tumor transcriptomes to infer treatment response. The first vignette is of a white male patient with bladder urothelial carcinoma with tumor mutational burden of 38.0 and a non-inflamed tumor microenvironment. Using the pre-treatment tumor biopsy transcriptome together with clinical and mutational features, Medea (GPT-4o) and Medea (Claude 3.7 Sonnet) predict response to atezolizumab. In this case, the Analysis and LiteratureReasoning pathways are concordant, concluding that the patient will respond based on COMPASS’s predicted responsiveness and immune signatures, including moderate-high cytotoxic T cell signal and interferon signaling, together with literature evidence linking treatment response to tumor microenvironment features and molecular subtype. GPT-4o and Claude 3.7 Sonnet used alone also predict response. The patient exhibits a partial response (RECIST [108]) after treatment.

In a second vignette, we consider a white female patient diagnosed with bladder urothelial carcinoma, tumor mutational burden of 14.0, and an inflamed tumor microenvironment. Using the pre-treatment tumor biopsy transcriptome together with clinical and mutational features, Medea (GPT-4o) and Medea (Claude 3.7 Sonnet) predict non-response to atezolizumab. In this case, the Analysis and LiteratureReasoning modules provide conflicting evidence: Analysis predicts non-response based on COMPASS’s outputs [36], whereas LiteratureReasoning emphasizes the literature associating higher tumor mutational burden with better response to immune-checkpoint inhibitors [109]. After multi-round discussions by MultiRoundDiscussion, Medea predicts non-response by prioritizing the microenvironment signature and by noting that tumor mutational burden is not a consistently reliable biomarker when used alone, as factors like immune cell composition can dominate response. In contrast, GPT-4o and Claude 3.7 Sonnet used alone both predict that the patient will respond to treatment. The patient has progressive disease (RECIST [108]), consistent with Medea’s prediction of non-response.

## Discussion

Medea is an agent that completes omics-based objectives for therapeutic discovery across biological contexts. Across three open-ended domains, cell type specific target nomination (2,400 analyses across five diseases), synthetic lethality reasoning (2,385 analyses across seven cell lines), and patient-level immunotherapy response prediction (894 patient analyses), Medea achieves strong performance while maintaining low failure rates and calibrated abstention. These results reflect a verification-aware design that specifies biological context, executes tool-grounded analyses with pre-run and post-run checks, evaluates and screens literature, and reconciles evidence or abstains when evidence is insufficient. In target nomination, Medea links candidates to disease-relevant cell types rather than tissue-level averages. In synthetic lethality, it integrates dependency signals with pathway context. In immunotherapy, it connects tumor microenvironment programs to personalized treatment response.

Context verification yields biologically specific interpretations. In rheumatoid arthritis, Medea ties targets to myeloid dendritic cells, naïve and effector-memory CD4^+^ *αβ* T cells, and naïve CD8^+^ *αβ* T cells rather than bulk tissue signals, improving accuracy within each cell type (e.g., by 28.9% in myeloid dendritic cells and 21.1% in effector memory CD4^+^ *αβ* T cells) while abstaining when the specified context is ambiguous. In follicular lymphoma, Medea nominates targets within disease-relevant B cell compartments and highlights pathways linked to germinal center biology, including B cell receptor signaling and immune synapse organization. Planning and execution checks further reduce failures by validating that the selected resources and model inputs match the specified context and data structure, avoiding common errors in agents [110].

The performance of AI agents on long-horizon tasks depends on verifying intermediate steps [111, 112]. In practice, tool-augmented agents often fail when tools time out, raise API exceptions, or return inconsistent outputs, which can trigger cascading errors and task abandonment. Medea adds explicit checks in planning and execution to detect tool malfunctions, apply structured retries, and update downstream reasoning. Because agentic training pipelines typically optimize for success trajectories and rarely expose models to tool failures [113], early mistakes in multi-step analyses can propagate and compound. In Medea, ablations show distinct roles for planning checks, execution diagnostics, and literature retrieval. A literature-only agent abstains in 77.6% of disease contexts and often withholds useful conclusions. An LLM-only agent abstains in 1.8% of analyses, but contributes the largest share of errors (69.2% on average across five diseases). The full Medea agent achieves the best performance with the lowest failure rate. In head-to-head comparisons with a biomedical agent, it fails to return outputs in 39.3% of rheumatoid arthritis cases due to preprocessing issues; among the remaining cases, Medea is 2.1 times more accurate.

Analyses of synthetic lethality and immunotherapy response further illustrate the benefits of context verification in Medea. For synthetic lethality, Medea surfaces lineage-aware genetic interactions whose joint inhibition is predicted to disrupt compensatory circuits, including pairs that couple DNA damage response or metabolic co-dependencies observed within specific cancer cell lines, with accuracy gains up to 22.0% across seven lines. For immunotherapy response, Medea stratifies patients by tumor mutational burden and tumor microenvironment features, and links response to antigen presentation, interferon signaling, and T-cell exhaustion programs. Prompt-robust behavior suggests that reconciliation steps reduce variance in conclusions that would otherwise arise from minor prompt changes [114].

Our study has limitations. First, the benchmarks rely on curated atlases, genetic dependency resources, and an immune checkpoint inhibitor naïve cohort, which may not capture the full diversity of tissues, lineages, or clinical settings; additional cohorts will be needed to test generalizability [36]. Second, performance for some tasks is judged with an LLM-based rubric and human adjudication [115]. Although we track abstention and failures, experimental follow-ups remain essential [116]. Third, tools can encode assumptions, including cell type granularity and batch structure. Medea mitigates this with context checks and execution diagnostics, yet mismatches can still bias outputs. Finally, the consensus stage relies on panel LLMs and confidence weighting, and correlated errors across panelists or adversarial prompts could influence results [117].

Future work can treat perturbations as the central object that links omics objectives to read-outs from omics datasets, enabling Medea to complete these objectives using experimental datasets rather than relying primarily on internet search. Adding measurements that resolve tissue context, including spatial transcriptomics [118–120] and disease-matched single-cell readouts [121, 122], can broaden the range of omics objectives supported by Medea. Looking ahead, extending evaluations to new biological domains will be important to establish general principles for AI agents.

## Online Methods

### 1 Overview of Medea

Medea is an omics AI agent for therapeutics discovery. Medea uses a global tool space of databases, APIs, machine learning models, and agents (Section 1.1). It consists of a ResearchPlanning module (Section 1.2), an Analysis module (Section 1.3), a LiteratureReasoning module (Section 1.4), and a MultiRoundDiscussion module (Section 1.5).

#### 1.1 Global tool space

Medea features a global tool space comprising 20 tools from 17 sources (13 databases/APIs and 4 machine learning models or agents). These tools are accessible to ResearchPlanning and Analysis modules throughout Medea’s runs.

**Retrieve Disease Targets** tool identifies and retrieves a disease’s protein targets through a two-step approach. Given a disease name, the tool first queries the EMBL-EBO API [38] to retrieve an Experimental Factor Ontology (EFO) ID for the disease. It then uses the EFO ID to interrogate the OpenTargets GraphQL API [39], extracting a list of associated protein targets based on specific evidence categories, such as genetic associations or approved therapeutic indications. By applying evidence-based filtering criteria, the tool outputs a curated list of protein targets.

**Load PINNACLE Embeddings** obtains pretrained cell type specific protein embeddings from PINNACLE [26]. PINNACLE integrates single-cell transcriptomics data with protein-protein interaction networks, resulting in 394,760 protein embeddings from 156 cell type contexts across 24 tissues. Given protein(s) and cell type context(s) of interest, this tool retrieves their corresponding cell type specific protein embeddings from PINNACLE. The final output is a dictionary containing context-specific protein embeddings, where keys are cell type contexts and values are cell type specific protein embeddings.

**Load TranscriptFormer Embeddings** retrieves cell type specific gene embeddings from TranscriptFormer [27], a single-cell transformer-based foundation model that models gene-gene interactions in single-cell transcriptomics data. The tool obtains precomputed Contextual Gene Embeddings (CGE) generated from single-cell atlases for rheumatoid arthritis [64], type 1 diabetes mellitus [123], Sjögren’s Syndrome [124], hepatoblastoma [67], and follicular lymphoma [125] (accessed via CELLxGENE [121]). Concretely, we perform TranscriptFormer inference on each cell type and disease combination using 16-bit mixed precision. As a result, we collect 2.1 million precomputed gene embeddings across 138 cell type and diseases. Given a list of gene names (official gene symbols or Ensembl IDs) along with disease and cell type contexts, the final output is a dictionary where keys are gene names and values are TranscriptFormer embeddings.

**Retrieve HumanBase PPI** retrieves tissue-specific protein-protein interaction networks, where nodes represent proteins and edges denote tissue-specific interactions annotated with confidence scores. Given a list of gene names and a tissue context, the tool first queries the Entrez API [126] to obtain the corresponding NCBI Entrez gene identifiers. It then calls the HumanBase RESTful API [37] to retrieve the relevant tissue-specific PPI network. From this network, an induced subgraph is generated based on the input gene list. The final output includes (i) a NetworkX [127] graph object representing the induced subgraph and (ii) a set of associations between the queried genes within the specified tissue context.

**HumanBase Co-Expression Analyzer** identifies genes that are co-regulated within a given tissue context. The tool accepts a gene list and a target tissue as inputs, and then queries the HumanBase API [37] to retrieve tissue specific co-expression interactions. Retrieved interactions undergo sequential quality control: raw correlation with coefficients ≤0.2 are excluded while those ≥0.7 are considered further. To ensure interpretability, the analysis does not rely solely on correlation strength: first, interactions lacking meaningful biological support are filtered out (evidence score ≤0.1); then, the top three most common evidence types behind the association are examined. The tool outputs a summary including co-expression network topology, network strength rating, ranked interactions with associated weights and supporting evidence, and a list of biological processes that are enriched in the co-expressed genes.

**HumanBase Protein Interaction Analyzer** identifies tissue-specific protein interactions by querying the HumanBase API, which integrates protein interaction data curated from BioGRID [128], IntAct [100], MINT [129], and MIPS [130]. Each protein pair is assigned a posterior probability weight based on experimental evidence. The tool retains edges with posterior probability ≥0.1 and prioritize those with ≥0.6 as high-confidence. Given a gene list, tissue name, and optionally the maximum number of interactions, the tool returns a summary of interaction types, confidence-weighted interaction pairs with supporting evidence, and a list of enriched biological functions.

**HumanBase Transcription Factor Analyzer** reconstructs tissue-specific regulatory networks for the genes of interest by analyzing transcription factor (TF)-gene relationships. Given a gene list, a target tissue, and optionally the maximum number of interactions, the tool queries the HumanBase API to retrieve a tissue-specific regulatory network derived from JASPAR motif predictions [37, 131]. TF-gene associations are identified through the scoring of binary motifs, with interactions filtered by evidence scores >0.1 to retain high-confidence predictions. The tool prioritizes TFs that may regulate the genes of interest in a given tissue context. The output includes the inferred TF-gene interactions, network connectivity scores, and enriched biological processes.

**HumanBase microRNA Target Analyzer** evaluates how microRNAs modulate gene expression in specific tissue environments. Given a set of genes and a tissue context, the tool queries the HumanBase API for microRNA target interactions curated from MSigDB (c3:MIR) [101], and constructs the corresponding regulatory network. The tool aggregates evidence types across interactions, and summarizes primary evidence categories. It returns a summary of observed microRNA targeting patterns, key regulators, and associated functional pathways.

**HumanBase Perturbation Analyzer** extracts tissue-specific perturbation relationships from a given list of genes and tissue context. The tool retrieves perturbation data from MSigDB (c2: CGP) [101] via the HumanBase API, and retains only high-confidence associations (interaction weight ≥ 0.6) that are supported by evidence (evidence score > 0.1). Each association is evaluated for functional relevance by aggregating and interpreting the underlying evidence types. The tool returns: (1) perturbation response of gene pairs from MSigDB (e.g., gene A knockdown affects gene B expression), ranked by HumanBase interaction confidence scores; and (2) Gene Ontology biological process terms annotated to the query genes, retrieved from HumanBase’s curated annotations.

**Enrichr Gene Enrichment Analyzer** uses Enrichr RESTful APIs [98] to perform functional enrichment analysis for a given gene pair across multiple curated libraries: WikiPathways 2024 [99], Reactome 2024 [40], MSigDB Hallmark 2020 [41], and the 2023 releases of GO Molecular Function and GO Biological Process [42, 132]. Given a pair of genes, the tool retains terms with Benjamini–Hochberg adjusted *P* ≤ 0.05 as “enriched” terms (reported by Enrichr). It also records Enrichr’s combined score for each gene–term association, and constructs a bipartite graph where nodes are the two genes and the set of “enriched” terms. Each gene-to-term edge is weighted by Enrichr’s combined score, which integrates Fisher’s exact P with a z-score (*c* = ln(*P*) × *z*) [98, 133] to capture both statistical significance and effect size. The tool then aggregates the combined scores and counts shared terms to assign an overall confidence level, and labels the putative interaction mechanism (e.g., signalling, metabolic, regulatory, complex) based on the biological context of those terms. It returns a structured report containing: (i) a summary of the inferred gene–gene relationship in natural language, (ii) an overall confidence based on aggregated combined scores and shared-term counts, and (iii) the five most significant shared pathways or GO terms.

**WikiPathways Co-Pathway Inspector** examines whether two genes participate in shared biological pathways by querying the community-curated WikiPathways 2024 Human corpus [99]. Given a pair of genes, the tool uses the Enrichr REST API to retrieve all human pathways that contain each gene in the WikiPathways 2024 Human corpus, and intersects these sets to pinpoint pathways in which the genes co-occur. For every shared pathway, it derives an interaction-context label (e.g., signalling cascade, metabolic chain) from edge metadata, and a confidence score of the interaction based on node proximity, edge evidence, and annotation depth. The output is a structured list of pathway names, their interaction classifications, and their confidence scores.

**Reactome Co-Pathway Analyzer** queries the Enrichr REST API to identify genes involved in shared molecular reactions based on the Reactome Pathways 2024 database [40]. The tool determines functional associations between the given genes by analyzing whether two genes participate in the same reaction events (e.g., phosphorylation, complex formation, direct binding, enzymatic activity). It takes a pair of genes as input and returns a summary of overlapping biochemical interactions, including the classification of the molecular mechanism and a confidence estimate.

**Hallmark Co-Pathway Analyzer** identifies functional relationships between genes based on shared involvement in cancer hallmark processes, using the MSigDB Hallmark 2020 collection [41] obtained via the Enrichr REST API. Given two genes, the tool evaluates whether they co-occur in curated hallmark pathways associated with oncogenic processes (e.g., apoptosis, proliferation, metabolic reprogramming, immune signaling, DNA repair, angiogenesis). Outputs are a summary of shared hallmark processes, mechanism classifications, and confidence annotations.

**GO Molecular Function Checker** evaluates whether two genes share similar molecular functions by querying curated annotations from GO Molecular Function 2023 [42, 132]. Given a gene pair, the tool accesses the GO molecular function annotations via Enrichr REST API calls, and searches for convergent functional assignments, mechanism categories, and relative confidence scores. The outputs are a list of shared functional terms, associated weights, and relevant mechanism labels drawn from GO’s standardized molecular vocabulary.

**GO Biological Process Checker** analyzes the enrichment of biological processes among the input genes by querying curated annotations from GO Biological Process (GO-BP) 2023 [42, 98, 132]. Through Enrichr REST API calls, the tool tests each gene separately and intersects the significant GO-BP terms (Benjamini–Hochberg adjusted *p* ≤ 0.05). For every shared term, the tool records the GO ID and Enrichr’s combined score. It then summarizes the overlap into an overall confidence (e.g., number of shared terms, distribution of combined scores). The tool’s outputs are a list of shared GO-BP terms (names and GO IDs), per-gene statistics (adjusted *p*-value, combined score), mechanism labels, and an overall confidence summary.

**Retrieve DepMap Correlations** obtains pairwise co-dependency statistics using Chronos gene-effect profiles from the DepMap 24Q2 CRISPR dataset [43]. The tool accesses preprocessed Chronos gene scores derived from CRISPR-Cas9 knockout screens across 1,320 cancer cell lines. For each pair of genes, it calculates the Pearson correlation coefficients [134] and the BH-adjusted *p*-values between the Chronos scores of the two genes [135]. A Chronos dependency score of a gene estimates the likelihood that the gene is essential in a cell line (0 indicates not essential, and −1 suggests comparable essentiality to the median of all pan-essential genes) [136].

**Generate COMPASS Predictions** applies the COMPASS model to predict immunotherapy response based on a patient’s transcriptomic profile [36]. Given a patient’s transcriptomic profile (TPM) and cancer type, the tool calls COMPASS to (1) predict the scores of 44 biologically-grounded immune concepts, capturing immune cell states, tumor microenvironment interactions, and signaling pathways, and (2) predict the likelihood of the patient responding to immune checkpoint inhibitors (ICIs).

**Search Scientific Literature** retrieves and synthesizes relevant scientific literature to answer the user query. It queries the Semantic Scholar API to collect candidate papers [47], and filters for methodological soundness and direct relevance. The resulting set of papers is fed into an Open-Scholar reasoning module [49], which synthesizes their key findings into a concise response with citations to address the query. The output is either a structured dictionary containing the literature-grounded summary with inline citations or, if the search yields nothing suitable, an explicit note that no sufficiently relevant study is found.

**HPA Biological Processes Analysis** performs functional characterization of individual genes by querying the Human Protein Atlas (HPA) API [44–46], which integrates expression data from 44 human tissues, 10 cancer cell lines, 8 blood cell types, and 7 regions of the brain. The tool processes these data to generate three outputs. The tool retrieves tissue-specific expression data (nTPM), experimentally validated Gene Ontology (GO) annotations, and protein-protein interaction (PPI) datasets. It maps the retrieved GO annotations to ten canonical biological categories (e.g., cell cycle, apoptosis, metabolism) to stratify the gene’s involvement in core cellular mechanisms. Additionally, the PPI datasets retrieved from the HPA API are used to identify functionally similar proteins, quantifying similarity via a Jaccard index of shared biological process profiles. Finally, for genes with tissue-specific expression data, the tool calculates fold changes between cancer cell lines and healthy tissues to quantify the magnitude of differential expression. The final report includes the categorized GO annotations, functionally similar proteins identified by overlapping biological processes, and calculated fold changes across cancer cell lines for the queried genes.

**HPA Comparative Expression Analysis** performs comparative gene expression analysis on 10 cancer cell lines against healthy tissues [44–46]. For each gene, the tool retrieves normalized Transcripts Per Million (nTPM) values via the Human Protein Atlas (HPA) API, and performs several comparative analyses, including fold-change calculation, statistical significance assessment, and expression level categorization. The tool reports per-tissue nTPM, per-cell-line nTPM, and tissue-vs-cell-line fold-changes. By default, the tool summarizes results from a panel of 10 cell lines (HeLa, MCF-7, A549, HepG2, Jurkat, PC-3, RH-30, SiHa, U-251 MG, Ishikawa). Expression levels are binned into analysis-defined ranges (from very low <0.1 to very high ≥50 nTPM). To prioritize candidates, the tool calculates a differential expression score based on magnitude (>3-fold = high, >2-fold = moderate) and flags highly upregulated targets, returning a summary report containing HPA metadata and fold-change metrics.

#### 1.2 ResearchPlanning module

Given free-form user input, consisting of a user instruction and an optional experimental instruction, the ResearchPlanning module iteratively transforms it into *in silico* research plans (Figure 2b). It leverages three module-specific tools: ResearchPlanDraft, ContextVerification, and IntegrityVerification. *ResearchPlanDraft* generates a multi-step research plan that explicitly states the objectives of the analyses, the selected tools, and the tools’ expected inputs, parameters, and outputs; *ContextVerification* validates each biological entity and parameter choice against tool knowledge bases to confirm data availability and compatibility; and *IntegrityVerification* checks the specificity, technical feasibility, and logical consistency of the research plan.

**ResearchPlanDraft** transforms free-form user input into a structured multi-step research plan, which consists of sequential computational analyses that address the research objective. Each step specifies the analysis objective, selected tool, required inputs and parameters, and expected outputs. The procedure is: (1) apply an LLM to distill the objectives of the research plan based on the given instructions; (2) perform LLM-augmented retrieval (RAG) to identify relevant tools and obtain their metadata from the global tool space; and (3) assemble the objectives, relevant tools, and tool metadata into a coherent, stepwise research plan.

**ContextVerification** ensures that all biological entities and parameter choices in the plan are compatible with the available tools and datasets from the global tool space. The procedure is: (1) apply an LLM to extract the names and parameters of the tools specified in the plan; (2) verify the tools’ availability in the global tool space; (3) invoke each tool’s internal context checker function, when available, to validate the parameter choices in the plan; and (4) if the required context is missing or invalid, use an LLM to recommend a set of tool-supported alternatives.

**IntegrityVerification** acts as the final audit of the completeness, coherence, and logical soundness of the research plan. An LLM judge, guided by a rubric-style systems prompt, evaluates: (1) clarity of the plan; (2) tool-use fidelity, ensuring that each tool’s usage aligns with the documented functionality and parameter specification; (3) parameter precision, confirming that the inputs follow the documentation and best practices; (4) hallucination risk, flagging elements unsupported by the provided data or tool instructions; and (5) logical coherence, verifying that the steps are sequential, build upon each other appropriately, and follow a clear step-by-step progression toward addressing the objective. Failure on any criterion triggers a revision request (i.e., returning a message that asks for another refinement cycle to improve the plan). The tool outputs an evaluation report that summarizes the unmet criteria and provides concrete recommendations for revision.

#### 1.3 Analysis module

The Analysis module translates the research plans from the ResearchPlanning module into well-documented, executable code for computational experiments. It coordinates four module-specific tools: CodeGenerator, AnalysisExecution, CodeDebugger, and AnalysisQualityChecker. *CodeGenerator* produces code snippets that implement the analyses specified in the research plan; *AnalysisExecution* runs the code in a sandboxed environment with resource and timeout controls, capturing standard output, error streams, and generated artifacts (e.g., tables, plots); *CodeDebugger* diagnoses runtime failures and revises code snippets using LLM-assisted debugging; and *AnalysisQualityChecker* performs post-execution evaluation, using structured criteria to assess correctness, reproducibility, parameter/provenance logging, and output informativeness against the user input and the research plan’s stated objectives. Altogether, the module operates through an autonomous iterative cycle, delivering executable code and *in silico* experimental results that address the user input and research plan without human intervention.

**CodeGenerator** uses a two-stage approach to generate executable code snippets for the analyses specified in the research plan. First, a ToolSelector component applies an LLM to identify the necessary tools and retrieve their associated metadata from the global tool space. Then, a separate LLM instance synthesizes code under a code-generation systems prompt that integrates the selected tools as outlined in the step-by-step research plan. Each generated code snippet undergoes a rubric-guided pre-execution check (e.g., syntax, interface compliance, parameter validity) using an LLM with a quality-check systems prompt; failures trigger bounded retries before returning a code snippet. This tool-aware code synthesis with iterative self-refinement is aligned with existing work on LLM tool-use and feedback-driven refinement [137, 138].

**AnalysisExecution** writes each code snippet to a temporary Python file and launches a Python subprocess with a 10-minute wait time to execute the code. Standard output and error streams are captured. Upon timeout, the tool captures the return code from the subprocess with the full traceback and logs attached for downstream analysis and debugging.

**CodeDebugger** is an LLM-based debugging tool. It analyzes the problematic code snippet and error messages, the user instructions, the research plan, and the tool specifications to identify the root cause. The CodeDebugger then generates corrected code snippets that address the errors while maintaining alignment with the research objectives and tool constraints, enabling iterative refinement until successful execution is achieved.

**AnalysisQualityChecker** evaluates the scientific validity and completeness of the successfully executed code using an LLM judge [115]. The tool analyzes the generated code and its execution outputs against the user instruction and research plan, examining code informativeness (i.e., outputs provide meaningful insights that are relevant to the research question), logical correctness (i.e., proper implementation of analytical workflows), and alignment with research objectives. The evaluation process uses structured LLM prompts that assess the quality of the analysis, and provides feedback in the tool’s output for iterative refinement if quality standards are not met.

#### 1.4 LiteratureReasoning module

The LiteratureReasoning module performs autonomous retrieval, relevance appraisal, and synthesis of peer-reviewed publications to produce literature-grounded analysis using three module-specific tools: LiteratureSearch, RelevanceVerification, and OpenScholarReasoning. *LiteratureSearch* executes structured searches across academic journal indices (e.g., Semantic Scholar [47], OpenAlex [48]) to collect candidate papers. *RelevanceVerification* performs study quality screening, scoring each paper for topical relevance and study type and returning a compact evidence profile per paper. *OpenScholarReasoning* synthesizes the screened set of papers into a literature-grounded report to address the research objective in user instruction.

**LiteratureSearch** conducts literature searches across Semantic Scholar [47] and OpenAlex [48] databases. The process involves: (1) using an LLM to extract domain-specific search terms from the user instruction; (2) conducting literature searches in parallel with Semantic Scholar and OpenAlex tools; (3) aggregating the retrieved papers and their metadata, and performing deduplication based on exact title matching and DOI comparison; and (4) returning a curated collection of paper abstracts, citation counts, publication metadata, and source attribution for downstream processing.

**RelevanceVerification** systematically assesses the relevance of a paper to the research objective using an LLM. For each retrieved publication, the tool analyzes its title, abstract, and metadata to assess study characteristics (e.g., species, disease, cell type context), and generates a binary relevance label and a detailed relevance summary.

**OpenScholarReasoning** synthesizes evidence from the curated collection of papers using the OpenScholar framework [49] with integrated reranking capabilities. The process involves: (1) initializing a BGE reranker model [139] to identify and prioritize the most informative passages via semantic similarity to the user instruction; and (2) using OpenScholar [49] to generate a literature-grounded report (with proper citation formatting) that addresses the user instruction.

#### 1.5 MultiRoundDiscussion module

The MultiRoundDiscussion module conducts a multi-round deliberation over the outputs of the Analysis module, LiteratureReasoning module, and backbone LLM to generate a consensus that addresses the user input. It adapts a ReConcile-style panel discussion [55] with standardized inputs, independent judgments, weighted reconciliation, iterative debate, and final synthesis.

Before deliberation, each module’s output is normalized into an evidence-grounded argument via a template with a fixed token budget and unified format. The deliberation has four main stages: (1) independent evaluation by each LLM panelist, (2) preliminary consensus among the panelists, (3) iterative discussion between the panelists until consensus is achieved, and (4) summarization of the panelists’ decision. In the first stage, three distinct LLMs independently evaluate each 300-word argument against the evaluation criteria (e.g., evidence strength, logical consistency, scientific rigor) to generate structured responses containing their preferred argument (i.e., from the Analysis module, LiteratureReasoning module, or backbone LLM), explanations, and confidence scores. The verdict of each LLM is saved in a JSON format. The three LLMs are configurable; by default, we use the backbone LLM, Gemini-flash-2.0 [140], and o3-mini [141]. In the second stage, preliminary consensus is determined by aggregating the verdicts from stage one via confidence-weighted voting using each panelist’s confidence score, which ranges from 0 to 1. If consensus is not achieved in stage two, the module initiates *R* (default is 2) rounds of debate. For each round, every panelist receives a debate prompt containing the arguments from each module and an audit trail summarizing the verdicts from the other panelists. Panelists reassess and update their own verdicts. When the panelists reach a consensus, the backbone LLM synthesizes the final response.

#### 1.6 Implementation details

##### Base LLM configuration.

Medea can be flexibly integrated with any LLM that supports function-calling and reasoning. Here, our experiments utilize Azure OpenAI GPT-4o (v2024-11-21; knowledge cutoff Sep 30, 2023) [142] and Claude Sonnet 3.7 (knowledge cutoff Feb 2025) [143] as backbone LLMs. Sampling temperatures are set to 0.4 for agent reasoning, 0.6 for tool invocations, and 1.0 for panel discussion modules; the remaining parameters are set to the default settings.

##### AgentLite framework.

Medea is developed using the AgentLite framework (v0.1.12) [144]. Each agentic module operates independently using the ReAct [145] architecture, performing an observe-act-reflect cycle for iterative tool-augmented reasoning. Individual modules maintain their own task-specific prompting schemas and memory modules for short-term trajectory tracking and long-term observation retrieval. As such, Medea’s architecture enables separation between reasoning logic, tool interaction, and task specialization.

### 2 Therapeutic target nomination

Nominating therapeutic targets requires reasoning about the candidate gene/protein in the disease and cellular contexts of interest. This is a biological question-answering task (Supplementary Table 1). We select five disease atlases from CELL×GENE [121, 146] for rheumatoid arthritis (RA) [64], type 1 diabetes mellitus (T1DM) [65], Sjögren’s syndrome (SS) [66], hepatoblastoma (HB) [67], and follicular lymphoma (FL) [68]. We process these disease atlases to identify disease-specific marker genes for each cell type (Section 2.1). With these marker genes and OpenTargets, we construct a benchmark dataset for therapeutic target nomination (Section 2.2).

#### 2.1 Single-cell disease atlas processing

We process disease atlases from CELL×GENE [121, 146] using the standard scanpy [147] pipeline. First, we remove cells with fewer than 200 expressed genes. To control cell-level quality across the disease atlases, we apply thresholds on mitochondrial read fraction and detected gene count. For T1DM, we use a mitochondrial threshold of 25% and a gene-count cap of 6,000; for RA, 20% and 800; for SS, 0% and 3,000; for FL, 15% and 5,000; and for HB, 8% and 8,000. Next, we filter out genes that are expressed in fewer than 3 cells and that are among the 10% least variable genes. Then, we normalize the total UMI counts to a scaling factor of 10,000 reads per cell, apply log normalization, and scale each gene to unit variance (and clip values exceeding 10 standard deviations). We also filter out genes with missing or duplicated NCBI IDs, Entrez IDs, or gene symbols. To evaluate the processing quality, we perform UMAP dimensionality reduction on the processed data. We visualize cell clustering with respect to known biological and technical metadata, including cell type, donor identity, and other dataset-specific annotations. These embeddings allow us to assess whether biologically meaningful groupings are preserved while identifying potential batch effects, indicated by clustering due to technical rather than biological factors. Finally, to identify disease-specific marker genes for each cell type, we perform differential expression analysis using a one-vs-all Wilcoxon rank-sum test. For each cell type within a given disease context, expression is compared against all other cells belonging to different disease statuses. This approach identifies genes that are specifically enriched in particular cell type and disease combinations.

#### 2.2 Dataset construction

Dataset construction follows a three-step procedure. First, we analyze the processed single-cell disease atlases from CELLxGENE [121] to identify disease-specific cell marker genes, which are significant differentially expressed genes (Wilcoxon rank-sum test with Bonferroni correction at *α* ≤ 0.05) within a cell type and disease context [148, 149]. Second, we collect 2,415 disease-associated genes/proteins from the Open Targets Platform [39]. We keep genes/proteins with either a genetic evidence score >0 [150] or ChEMBL evidence score >0 [70]. Third, we define ground-truth cell type specific disease targets as the genes/proteins satisfying the criteria from both steps 1 and 2. For each cell type context, we sample one target (i.e., positive gene/protein) and four negative genes/proteins that do not meet the criteria to form five-gene candidate lists. Prompts are paraphrased with o3-mini-0131 (temperature = 1.0) under a “biologist” role. Using three random seeds, we generate 20 samples per cell type per disease, producing 2,400 analyses: 420 for RA, 600 for T1DM, 360 for SS, 180 for HB, and 840 for FL (Figure 3b; Figure 4b; Supplementary Table 1).

### 3 Synthetic lethality prediction

Predicting synthetic lethality (SL) requires reasoning about genetic dependencies in a certain cellular context to infer whether perturbing two genes together reduces cellular viability substantially more than perturbing either gene alone [94, 95]. This is an open-ended reasoning task.

#### 3.1 Dataset construction

We curate experimentally-validated (via combinatorial genetic screening [96]) SL gene pairs and matched negative gene pairs. Gene pairs are collected from seven cell lines (six tumor-derived and one non-tumorigenic control from distinct genomic contexts) to capture diverse genomic dependencies [151]: *KRAS* gain-of-function mutations (MDAMB231, A427, A549), *PIK3CA* gain-of-function mutations (MCF7, CAL33), *TP53* mutations (MDAMB231, CAL27, CAL33), and no such mutations (MCF10A) [96]. These cell lines represent contexts in which cancer therapeutic targets have been extensively characterized using CRISPR screens [152]. For each positive gene pair in a specific cell line, (gene_*a*_, gene_*b*_, cell_*x*_), two negative gene pairs are generated via random substitution from a pool of 9,987 reported non-SL pairs, (gene_*a*_, gene_*c*_, cell_*x*_) and (gene_*b*_, gene_*d*_, cell_*x*_). The substituted genes (gene_*c*_ and gene_*d*_) are experimentally-validated to not have a synthetic lethal interaction with the positive genes (gene_*a*_ and gene_*b*_) in any of the cell line contexts [96]. This strategy preserves structural similarity to the positive pairs while ensuring non-lethality. With these triplets of gene pairs and cell line context, we construct an open-ended reasoning benchmark for context-specific SL prediction (Figure 5b; Supplementary Table 1). Prompts are paraphrased with o3-mini-0131 (temperature = 1.0) under a “biologist” role. The dataset contains 1,855 analyses.

### 4 Immunotherapy response prediction

Predicting personalized immunotherapy response requires reasoning about each patient’s clinical features, genomic profile (e.g., tumor mutational burden), and tumor microenvironment [104, 106, 107]. This is an open-ended reasoning task on multimodal inputs.

#### 4.1 Dataset construction

We construct the dataset using the IMvigor210 cohort (*N* = 298) [105], a single-arm phase II study of anti-PD-L1 atezolizumab in patients with metastatic urothelial carcinoma. For each patient, we create a user instruction containing the patient’s clinical metadata: tumor mutational burden (TMB) [153], demographics (sex, race), tissue source, and treatment regimen (Figure 6b; Supplementary Table 1). We create two additional prompt templates that contain the user instruction and an experiment instruction to analyze the patient’s transcriptomic profile (Figure 6c). Prompts are paraphrased with o3-mini-0131 (temperature = 1.0) under a “clinician” role. Using three random seeds, we generate 2,682 analyses (3 seeds × 298 patients × 3 prompt templates).

### 5 Evaluation of model outputs

We provide details about model evaluation, performance metrics, and statistical analyses.

#### 5.1 LLM judge

We use an LLM-as-a-Judge (or LLM judge) framework [115, 154] to assess model outputs. Notably, we incorporate mechanisms for selective prediction [155, 156] to handle three special cases: (1) *Abstain*, where the model explicitly admits insufficient evidence or inconclusive analyses [157]; (2) *Failed*, where the model does not return any substantive analysis; and (3) *None*, where the model systematically evaluates and rejects all candidates based on context-specific criteria.

##### Multiple choice evaluation.

For therapeutic target nomination (Section 2; Supplementary Figure 1) and immunotherapy response prediction (Section 4; Supplementary Figure 2), the LLM judge examines the model output to classify the prediction as one of the predefined categories. For therapeutic target nomination, the LLM judge either provides the target gene name or classifies the output into one of three categories (Supplementary Figure 1): *Abstain*, *None*, or *Failed*. For immunotherapy response prediction, the LLM judge classifies the output into one of four categories (Supplementary Figure 2): *R* (responder), *NR* (non-responder), *Abstain*, or *Failed*.

##### Open-ended reasoning.

The LLM judge uses structured prompt templates that provide label definitions with examples [158]. This approach mirrors established methodologies for research claim verification, where the models must discern whether the evidence supports or refutes a hypothesis [159]. For synthetic lethality prediction (Section 3), the LLM judge evaluates the model’s open-ended reasoning trace to classify it as one of four categories (Supplementary Figure 3): (1) *Synthetic lethality*, (2) *Non-SL*, (3) *Abstain*, or (4) *Failed*.

#### 5.2 Model evaluation

Medea is benchmarked against five large language models (LLMs) and one biomedical agent.

##### Large Language Models (LLMs).

We evaluate Medea against five state-of-the-art LLMs: GPT-4o [142], o1-mini [160], o3-mini [141], DeepSeek-R1:671B [161], and Claude-3.7-Sonnet [143].

##### Biomedical AI Agents.

CellVoyager is designed to reproduce single-cell analyses from scientific papers [21]. We evaluate its ability to nominate therapeutic targets for rheumatoid arthritis [26].

#### 5.3 Accuracy metric

We adopt the selective prediction evaluation protocol [162, 163]. We define accuracy as the proportion of analyses for which the model output contains a definitive and correct answer (Section 5.1). Formally, given *N*_*total*_ analyses, the accuracy (or selective risk) is calculated as:

(1) $$\text{Accuracy}=\frac{1}{N_{\text{total}}-N_{\text{abstain}}}\sum_{i=1}^{N_{\text{total}}}1\left\{{\hat{y}}_{i}=y_{i}\wedge {\hat{y}}_{i}\neq \text{Abstain}\right\},$$

where **1**{·} is the indicator function that equals 1 when the predicted category matches the ground-truth label (case-insensitive) and 0 otherwise, and *N*_*abstain*_ denotes the total number of analyses classified as *Abstain* (Section 5.1). *Abstain* cases occur when models explicitly acknowledge insufficient evidence or uncertainty in their reasoning. As abstentions are not incorrect predictions, we exclude them from accuracy calculations, decoupling correctness from coverage [155]. We additionally report the abstention rate *r*_*abstain*_ = *N*_*abstain*_*/N*_*total*_.

#### 5.4 Statistical significance testing

We use McNemar’s test [71] to evaluate the statistical significance between two models’ performance. We construct a 2 × 2 contingency matrix. Each entry *n*_*ij*_ indicates the number of analyses in which Medea’s correctness is $i\in \left\{0,1\right\}$ and the other model’s correctness is $j\in \left\{0,1\right\}$, where 0 represents correct and 1 represents incorrect. *n*_01_ denotes the number of analyses where Medea is correct but the other model is incorrect. *n*_10_ denotes the number of analyses where Medea is incorrect but the other model is correct. We exclude analyses where either model abstains (Section 5.1). The McNemar’s test statistic is calculated as:

(2) $$\chi^{2}=\frac{{\left(n_{01}-n_{10}\right)}^{2}}{n_{01}+n_{10}}$$

using the implementation from the statsmodels package [164].

## Data Availability

The model checkpoints, datasets, and Medea’s tools are available on Hugging-Face at https://huggingface.co/datasets/mims-harvard/MedeaDB.

## Code availability.

The documentation of Medea is available at https://medea.openscientist.ai. The implementation of Medea and the code to reproduce the analyses in the paper are available on GitHub: https://github.com/mims-harvard/Medea.
